# Effects of Angiotensin-Converting Enzyme Inhibition on Circulating Endothelial Progenitor Cells in Patients with Acute Ischemic Stroke

**DOI:** 10.1155/2018/2827580

**Published:** 2018-04-26

**Authors:** Monika Gołąb-Janowska, Edyta Paczkowska, Bogusław Machaliński, Dariusz Kotlęga, Agnieszka Meller, Krzysztof Safranow, Michał Maj, Przemysław Nowacki

**Affiliations:** ^1^Department of Neurology, Pomeranian Medical University, Szczecin, Poland; ^2^Department of General Pathology, Pomeranian Medical University, Szczecin, Poland; ^3^Department of Biochemistry and Medical Chemistry, Pomeranian Medical University, Szczecin, Poland

## Abstract

**Background:**

Therapeutic neovascularization might represent an important strategy to salvage tissue after ischemia. Circulating bone marrow-derived endothelial progenitor cells (EPCs) were previously shown to augment the neovascularization of ischemic tissue. Angiotensin-converting enzyme inhibitors (ACEIs) might modulate EPC mobilization. We evaluated populations of circulating stem cells and early EPCs in acute ischemic stroke (AIS) patients and the effect of ACEI on circulating EPCs in these patients with respect to aspects of stroke pathogenesis.

**Methods:**

We studied 43 AIS patients (group I), comprising 33 treated with ACEI (group Ia) and 10 untreated (group Ib). Risk factor controls (group II) included 22 subjects. EPCs were measured by flow cytometry.

**Results:**

In AIS patients, the number of circulating stem cells and early EPCs upon admission was similar to that in control group individuals. There were no significant differences in the numbers of stem cells and early EPCs over subsequent days after AIS. There were also no significant differences in stem cell and early EPC numbers over the first 3 days between group Ia and group Ib. However, on day 7, these numbers were significantly higher in group Ib than in group Ia (*p* < 0.05). In AIS patients chronically treated with ACEI, there was a negative correlation between CD133^+^ cell number and neurological deficit on the first, third, and seventh days (*p* < 0.005).

**Conclusions:**

An increased number of circulating stem cells and early EPCs were not observed in stroke patients chronically treated with ACEI. In patients chronically treated with ACEI, a significant correlation was observed between decreased neurological deficit and higher levels of CD133^+^ cells; this could be due to the positive influence of these cells on the regeneration of the endothelium and improved circulation in the ischemic penumbra.

## 1. Introduction

Stroke is the third leading cause of death and the most common cause of permanent disability in adults worldwide. Following acute ischemic stroke (AIS), a complicated cascade of biochemical events takes place that involves inflammation, neuronal necrosis, disruption of the blood-brain barrier, and neurological dysfunction [1–4].

The balance between endothelial injury and repair and the turnover of endothelial cells are the major determinants of vascular integrity maintenance. An imbalance represents a key step in atherosclerosis. The number of circulating endothelial progenitor cells (EPCs) can be the result of the ability of the bone marrow to mobilize them. Some of these cells can be destroyed in the circulation due to the various factors (e.g., hypertension, high cholesterol levels, and inflammation); the rest are incorporated into the damaged endothelium. In addition, assessing endothelial damage is important when evaluating EPC levels [5].

Endothelial progenitor cells (EPCs) are released from the bone marrow to the peripheral blood and participate in endothelial cell repair and regeneration [1, 5]. Such EPCs most likely coexpress specific endothelial and progenitor markers such as CD34, CD133, and vascular endothelial growth factor receptor (VEGF-R) [6]. As shown in animal and human models, EPCs contribute to neovascularization and reendothelialization [7]. Clinical studies have revealed that cardiovascular diseases are associated with a dysfunction in EPCs [8, 9] and that the number of circulating EPCs correlates positively with clinical outcome [10–13]. Although EPCs might be a potential marker of vascular function in cardiac disease, few studies have delved into the contribution of EPCs to clinical outcome after AIS. Furthermore, the results are conflicting; some studies have reported lower EPC counts in patients with acute stage ischemia compared to that in controls [14–16], whereas other studies have reported the opposite [17–21].

In vitro and clinical studies have shown that drugs used in the treatment of cardiovascular diseases, such as angiotensin-converting enzyme inhibitors (ACEIs), have beneficial effects on EPC mobilization [22, 23]. Experimental studies have also revealed that ACEIs can attenuate the development of atherosclerosis-related diseases independent of their vasodilating and hypotensive effects, and this attenuation might be associated with the modulation of EPC mobilization [24]. In patients with coronary artery disease (CAD), ACEIs have been shown to improve prognosis, although the underlying mechanisms are not fully understood [25]. ACEIs increase the expression of many signaling molecules including vascular endothelial growth factor (VEGF) [24, 26]. These molecules are released into the circulation from the ischemic myocardium and act on the bone marrow to promote the release of EPCs [27].

Taken together, EPC levels are inversely correlated with various risk factors and positively correlated with astrocytes, EPO, and angiogenic T-cells, whereas the relationship between ACEIs and EPCs remains controversial. Despite the above data suggesting both inhibitory, negative effects and enhancing, positive outcomes, it remains unclear whether EPCs play a positive role in AIS [28].

Accordingly, we evaluated the populations of circulating stem cells (CD133^+^) and early EPCs (CD133^+^/VEGFR2^+^) in AIS patients, in addition to the functional, chemotactic effect of ACEIs on circulating EPCs in these patients, taking into account aforementioned aspects of stroke pathogenesis.

## 2. Materials and Methods

### 2.1. Patient Study Group

We prospectively studied 43 consecutive patients with AIS (group I); 33 were treated with an ACEI (group Ia), and 10 were untreated (group Ib). These individuals were admitted to the Department of Neurology, Pomeranian Medical University in Szczecin. All patients were enrolled in the study within the first 24 hours after the onset of stroke.

The number of patients studied on day 3 was 37 (27 for group Ia, 10 for group Ib); no sample was available in cases of defective blood sampling (*n* = 6). The number of patients studied on day 7 was 32 (25 for group Ia, 7 for group Ib); no sample was available from the remaining patients due to death (*n* = 2), early discharge (*n* = 4), or defective blood sampling (*n* = 5).

Exclusion criteria were as follows: a previously modified Rankin scale [29] score higher than 2; a National Institute of Health Stroke Scale (NIHSS) [30] score of 0; current history of acute inflammatory disease, chronic inflammatory disease, neoplastic disease, and renal failure; and lack of blood sample processing within 2 hours of collection (the predefined time window for obtaining reliable results). Because our laboratory could process the blood samples only during working days, we excluded patients admitted during the weekend as the sample could not be processed within 2 hours. Risk factor controls (group II) included 22 subjects matched by age, gender, and traditional vascular risk factors (see subsequent sections).

The study adhered to the principles of the Declaration of Helsinki, and approval was obtained from the Local Research Ethics Committee (Szczecin, Poland). Moreover, written informed consent was obtained before patient involvement.

### 2.2. Clinical Data

We recorded the following data from each patient: demographics (age and sex); presence of traditional vascular risk factors including high blood pressure, diabetes mellitus, hypercholesterolemia, CAD, atrial fibrillation, peripheral artery disease, smoking habits, alcohol abuse, previous transient ischemic attack, and previous cerebral infarction; and treatment with any ACEI before the onset of stroke and during admission.

#### 2.2.1. Risk Factors

Regarding hypertension, patients were diagnosed based on their history, medical records, and drugs taken. Newly detected arterial hypertension was diagnosed when repeated tests showed a systolic blood pressure ≥ 140 mmHg and/or a diastolic blood pressure ≥ 90 mmHg [31]. The criteria for diagnosing type 2 diabetes were patient history, medical records, and drugs administered. Confirmation of newly diagnosed diabetes in patients was based on HbA1c ≥ 6.5% at admission [32]. Hyperlipidemia (serum cholesterol concentration > 190 mg/dL, LDL cholesterol > 115 mg/dL, or serum triglyceride concentration > 150 mg/dL) was assessed as defined previously [33]. Coronary artery disease (CAD) was defined as prior myocardial infarction, angina pectoris, percutaneous coronary intervention, or coronary artery bypass surgery. Following CC/AHA/ESC clinical guidelines, atrial fibrillation was defined as the absence of P waves in the ECG, with the isoelectric line being replaced by irregular high-frequency oscillations (f waves), and the wholly irregular ventricular response; this was based on ECG results during admission or from previous medical reports [34]. Peripheral arterial disease included a previous history of intermittent claudication, arterial thrombosis, and percutaneous or surgical intervention in the thoracic, abdominal aorta, or lower extremity vessels. Current smoking habits were also considered. Moreover, alcohol abuse was taken into account if the consumption of ethyl alcohol by men exceeded 40 g per day (>4 standard doses of spirits) and 20 g per day (>2 standard doses) for women [35].

Body mass index was also assessed; a classification of overweight was indicated by a BMI of 25.0–29.9, and obesity was determined with a BMI > 30. Finally, a history of cerebral vascular episodes was considered. For this, we analyzed stroke etiological subtypes according to the SSS-TOAST classification [36] and severity of the neurological deficit on admission (NIHSS score). Improvement of neurological status was defined as a decrease in the NIHSS score percentage between admission and the seventh day after stroke onset. In each case, the stroke had been precisely documented by computer tomography.

### 2.3. Laboratory Measurements

EDTA-anticoagulated peripheral blood (PB) samples (two 2.7 mL specimens) were drawn within 24 hours of stroke onset, as well as on days 3 and 7, from stroke patients, and once from control subjects. The absolute numbers of leukocytes and lymphocytes in PB were determined using an automatic cell counter (Cell-Dyn 3500, Abbott Diagnostics, Santa Clara, CA, USA). High-sensitivity CRP (hsCRP) was assayed using a latex-enhanced immunonephelometric assay on a BN II analyzer (Dade Behring, Newark, DE), whereas fibrinogen was measured on this device by immunoassay. The full population of peripheral blood nucleated cells (PBNCs) was obtained after the lysis of red blood cells using 1 × BD Pharm Lyse Buffer (Pharmingen, BD Biosciences, San Diego, CA, USA).

### 2.4. Flow Cytometry

The full population of PBNCs was obtained after the lysis of erythrocytes using BD Pharm Lyse Buffer (Pharmingen, BD Biosciences, San Diego, CA, USA). For flow cytometry, 1 million NCs were resuspended in 100 *μ*L of phosphate-buffered saline (PBS). Immunofluorescence cell staining was performed using a phycoerythrin- (PE-) conjugated anti-CD309 (VEGFR-2/KDR) antibody (Pharmingen, BD Biosciences, San Jose, USA; clone 89106) and an allophycocyanin- (APC-) conjugated anti-CD133 antibody (Miltenyi Biotec, Auburn, CA, USA; clone AC133). Samples stained with appropriate isotype control antibodies, specifically IgG1-PE (Pharmingen, BD Biosciences, San Jose, USA; clone MOPC-21) and IgG1-APC (Miltenyi Biotec, Auburn, CA, USA), were prepared in parallel and served as negative controls. After 20 min incubation on ice, the cells were washed twice in PBS and resuspended in 1% paraformaldehyde. Cell fluorescence was measured, and data were analyzed using an LSRII flow cytometer (BD Biosciences, San Jose, CA, USA) with BD FACSDiva software. Typically, 2 × 10^5^ events were acquired to determine the proportions of examined subpopulations within the PBNCs. Using this approach, populations of circulating stem cells (CD133^+^) and early EPCs (CD133^+^/VEGFR2^+^) were analyzed.

### 2.5. Statistical Methods

The primary objective of this trial was to investigate the alternative hypothesis that ACEI treatment is associated with higher populations of circulating stem cells (CD133^+^) and early EPCs (CD133^+^/VEGFR2^+^) in AIS patients (the biological null hypothesis assumed that ACEI treatment does not increase the number of EPCs in AIS individuals).

There were two main types of variables assessed, namely, quantitative and qualitative.

Because the distribution of quantitative variables was not normal and skewed (Shapiro–Wilk test), the data were compared between the groups using the nonparametric Mann–Whitney *U* test. The results of qualitative variables were presented as a number (percentage). Data were compared between the groups using the *χ*^2^ test. Correlations between quantitative variables were studied by assessing Spearman's rank correlation coefficient (Rs). Multiple linear regression was used to check whether acetylsalicylic acid treatment, plasma hsCRP, and plasma fibrinogen were independent factors associated with the logarithmically transformed number of CD133^+^ cells or EPCs in group I patients.

All statistical tests were two-tailed, and *p* < 0.05 was considered to indicate a statistical significance. All statistical analyses were performed with STATISTICA 10 software.

## 3. Results

Table 1 displays the baseline characteristics of groups I and II. There were no significant differences in terms of these variables between the assessed groups with the exception of higher plasma hsCRP in AIS patients. The numbers of CD133^+^ and CD133^+^/VEGFR2^+^ cells in groups I and II were also similar (*p* > 0.05; Table 2).

In AIS patients, circulating stem cells (CD133^+^) were decreased in the first week, but there were no significant differences in their numbers on subsequent days after AIS (CD133^+^_1 = 0.0325 ± 0.07, CD133^+^_3 = 0.0177 ± 0.01, and CD133^+^_7 = 0.0182 ± 0.02; *p* > 0.05). Early EPCs (CD133^+^/VEGF-R2^+^) were also decreased during the first week, and there were no significant differences in their numbers at subsequent days after AIS (CD133^+^/VEGF-R2^+^_1 = 0.0099 ± 0.022, CD133^+^/VEGF-R2^+^_3 = 0.0049 ± 0.004, and CD133^+^/VEGF-R2^+^_7 = 0.0048 ± 0.004; *p* > 0.05).

Group Ia consisted of 33 patients treated with ACEI. All patients were treated exclusively with perindopril at 5 mg per day prior to stroke and a stable dose during admission.

As shown in Table 3, group Ia had significantly higher blood hsCRP (*p* = 0.0223) and fibrinogen (*p* = 0.0478) levels than group Ib.

**Figure 1:**
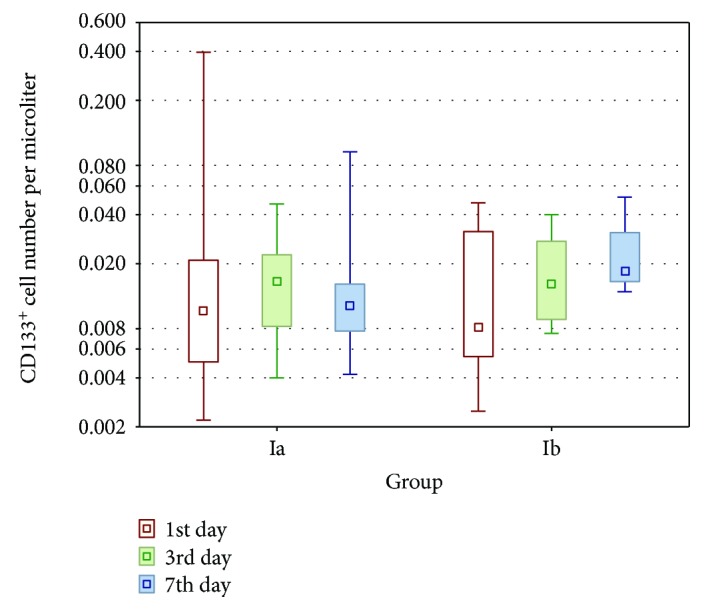
The populations of circulating stem cells (CD133^+^) in groups Ia and Ib on the 1st, 3rd, and 7th days. On the 7th day, the number of cells was significantly higher in group Ib (*p* < 0.05, Mann–Whitney *U* test).

**Figure 2:**
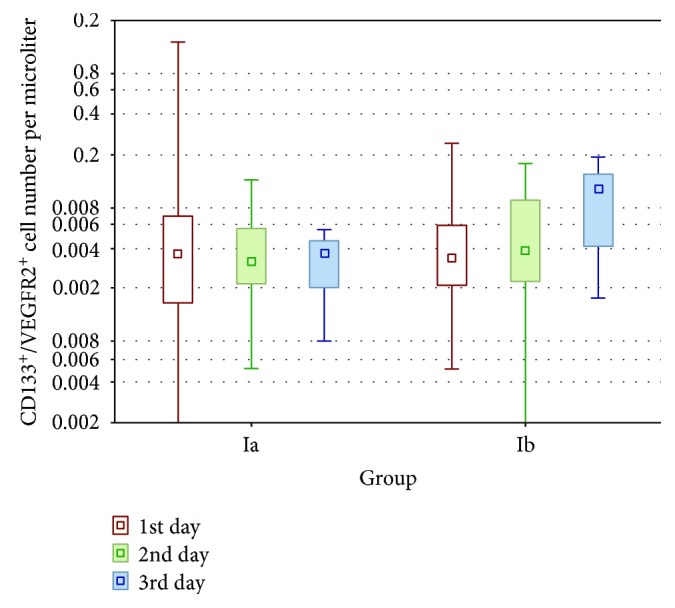
The populations of early EPCs (CD133^+^/VEGFR2^+^) in groups Ia and Ib on the 1st, 3rd, and 7th days. On the 7th day, the number of cells was significantly higher in group Ib (*p* < 0.05, Mann–Whitney *U* test).

In group Ia, the number of circulating stem cells (CD133^+^) and the number of early EPCs (CD133^+^/VEGFR2^+^) were highest on the first day and then decreased on days 3 and 7; however, the differences between the numbers of circulating cells at these time points were not significant (*p* > 0.05). There were only small differences between the number of CD133^+^ cells and the number of CD133^+^/VEGFR2^+^ cells in group Ib during observation (*p* > 0.05; Tables 4 and 5). On days 1 and 3, the numbers of CD133^+^ and CD133^+^/VEGFR2^+^ cells were not significantly different between group Ia and group Ib (*p* > 0.05; Figures 1 and 2). On day 7, the numbers of stem cells (CD133^+^) and early EPCs (CD133^+^/VEGF-R2^+^) were significantly higher in group Ib than in group Ia (*p* = 0.0122 and *p* = 0.0041, respectively; Figures 1 and 2).

In group Ia, there was a negative correlation between CD133 ^+^ cell number on day 7 and neurological deficit, as assessed by NIHSS, on day 1 (*N* = 25, Rs = −0.42, *p* = 0.0377), day 3 (*N* = 25, Rs = −0.45, *p* = 0.0251), and day 7 (*N* = 25, Rs = −0.58, *p* = 0.0025). Additionally, in group Ia, there was a positive correlation between CD133^+^ and CD133^+^/VEGF-R2^+^ cell numbers and concentration of hsCRP on day 1 (*N* = 32, Rs = 0.42, *p* = 0.0158 and *N* = 32, Rs = 0.53, *p* = 0.0018, resp.). Moreover, the number of early EPCs (CD133^+^/VEGF-R2^+^) on day 3 was positively correlated with concentration of LDL cholesterol (*N* = 27, Rs = 0.39, *p* = 0.0452).

Multiple linear regression analysis showed that ASA dose and the levels of hsCRP and fibrinogen, which were significantly higher in group Ia, were not independent factors significantly influencing the numbers of circulating stem cells (CD133^+^) and early EPCs (CD133+/VEGF-R2^+^), and the only independent factor significantly associated with the lower number of these cells on day 7 after AIS was ACEI treatment (*p* = 0.0290 for CD133^+^ and *p* = 0.0019 for CD133+/VEGF-R2^+^).

## 4. Discussion

This study comprised four major findings. First, in AIS patients, the numbers of circulating stem cells (CD133^+^) and early EPCs (CD133^+^/VEGFR2^+^) upon admission were similar to those in control individuals without AIS; however, the risk factors were similar. Second, there were no significant changes in the numbers of stem cells (CD133^+^) and early EPCs (CD133^+^/VEGF-R2^+^) at subsequent days after AIS. Third, chronic treatment with ACEIs does not increase the number of circulating stem cells (CD133^+^) and early EPCs (CD133^+^/VEGFR2^+^) in AIS, and in fact, it is an independent factor associated with lower numbers of these cells on day 7 after AIS. Lastly, in AIS patients chronically treated with an ACEI, the number of CD133^+^ cells was negatively correlated with neurological deficit.

Ghani et al., Chu et al., and Zhou et al. reported a reduction in EPCs among patients with cerebrovascular diseases, compared to that in healthy controls [14–16]. However, these data are inconsistent as other authors found higher EPC counts in stroke patients than in controls [17, 21]. Yip et al. [18] showed that the levels of circulating EPCs (CD31^+^/CD34^+^, CD62E^+^/CD34^+^) and (KDR^+^/CD34^+^) were significantly higher among patients with AIS than in the at-risk control subjects. Paczkowska et al. [19] reported a significantly higher percentage of early EPCs only in hemorrhagic stroke patients as opposed to those with AIS. Our study shows that the number of circulating EPCs is similar between acute stroke patients and risk factor control cases. A number of variables such as age or distribution of risk factors, time from stroke onset to blood collection, EPC definition, EPC measurements, and statistical methods performed in different papers might account for these discrepancies.

After ischemic injury, the release of cytokines and trophic factors might induce an increase in the production and mobilization of EPCs [37]. This occurs in patients with acute coronary syndrome and acute ischemic stroke, with a peak in EPC counts and vascular endothelial growth factor (VEGF) levels occurring on the seventh day after the ischemic event [16, 38–40]. However, one study reported stable EPC counts, whereas another reported the intermittent release of EPCs after ischemic stroke [15, 17]. In our study, we confirmed stable levels of CD133^+^ and CD133^+^/VEGFR2^+^ cells in AIS.

In previous studies involving patients with myocardial infarction, a higher number of EPCs were associated with better prognosis, increased myocardial salvage, and more collateral in the ischemic zone [41, 42]. However, the impact of the number of circulating EPCs on clinical outcomes after stroke in men remains uncertain. Previous studies have shown that low circulating EPC levels are independently predictive of severe neurologic impairment and major adverse clinical outcomes in patients with AIS [18]. According to another study regarding patients after AIS, good neurological and functional outcome, reduced infarct growth, and neurological improvement have been associated with the increase of EPCs [43]. Probably, in addition to the severity of the stroke, the number of circulating EPCs is influenced by other factors such as age, comorbidities, and drugs used [44, 45]. However, the increased number of circulating EPCs is thought to play an important role in endogenous vascular repair and in the maintenance of endothelial integrity in the adult brain after ischemic stress [46, 47].

EPCs activated by perindopril could contribute to ongoing endothelial repair by providing a circulating pool of cells that can be home to injured parts of the artery or replace dysfunctional endothelial cells [8]. Additionally, EPCs, once mobilized into the peripheral circulation, penetrate the infarct and are involved in the formation of new vessels [48], which then improves blood supply in the ischemic penumbra, finally leading to a decreased infarction area after AIS [49]. The significant activation of EPCs might be associated with the different effects of ACEIs themselves, including inhibiting bradykinin degradation, increasing cGMP in vascular endothelial cells, restraining renin-angiotensin system activity, and decreasing the production of oxygen free radicals, which leads to sensitized NO synthase and increased NO release. All of these effects can, in turn, induce EPC mobilization from the bone marrow [50].

We observed that chronic treatment with ACEIs does not additionally increase the number of circulating stem cells (CD133^+^) and early EPCs (CD133^+^/VEGFR2^+^) in AIS. In contrast, our results demonstrate the existence of a statistically significant relationship between the number of CD133^+^ cells and neurological deficit, as assessed by NIHSS. On the seventh day after ASI, a negative correlation between CD133^+^ and NIHSS score was found, which could be the result of improved circulation in the ischemic penumbra mediated by a positive influence of CD133^+^ cells on the endothelial regeneration of damaged vessels or even on the formation of new vessels. Consequently, this mechanism could promote a reduction in the neurological deficit. The interpretation of the results from the first and third days is difficult. We can assume that the severity of the neurological deficit is first correlated with neural tissue necrosis and compensative mechanisms are not sufficient.

In terms of prognosis after AIS, ACEIs seem to be a promising option for the treatment of AIS and for the primary prevention of cerebrovascular events. Many factors, including growth factors, chemokines, proinflammatory cytokines, chemokines, hormones, and lipid-lowering and antidiabetic drugs, have been regarded as important factors involved in EPC mobilization [51].

Circulating hsCRP represents a potential independent predictor of vascular damage [52]. Initially proposed as a biomarker, hsCRP was subsequently suggested as a player in atherogenesis [53], although its role has not been definitely defined [54]. Verma et al. demonstrated that EPCs incubated with human recombinant CRP, at concentrations known to predict adverse vascular outcomes, exhibited decreased survival and increased apoptosis. Moreover, the reduction of EPCs appears to be hsCRP dose-dependent [55, 56].

Fibrinogen is an inflammatory marker and is closely related to atherosclerosis [57–59].

Indeed, Mandraffino et al. [60] showed a strong correlation between inflammatory markers (including CRP and fibrinogen) and EPC levels in several different clinical settings (smokers, arthritis patients, and hypertensive patients with and without left ventricular hypertrophy); here, it was stated that “CD34+ cell count change at T1 appeared to be mainly correlated with fibrinogen reduction (Rs = −0.625, *p* < 0.001).” This group also proposed different mechanisms through which inflammation (including mediators, cytokines, receptors, and microRNAs), and also RAAS modulation (including drug intervention) might affect the EPC number [53–58]. Moreover, the relationships between fibrinogen and EPCs have also been evaluated in elderly individuals, where inflammatory markers appear to lose their predictive ability [59]. Our study showed that serum fibrinogen and hsCRP levels were significantly higher in group Ia, although multiple linear regression did not reveal them to be independent factors affecting EPC mobilization.

Our study has several limitations. First, it lacks the detection of appropriate cell surface markers specific for the identification of EPCs in the field of EPC research. The conflicting results might be due to different methodologies used to identify circulating EPCs. Second, the study was performed using a small sample size of 43 subjects, and the results require further confirmation in larger cohorts. Third, the number of circulating EPCs is influenced by many factors and the basal levels of EPCs from each patient are not known, specifically, steady-state levels and levels immediately before the acute vascular event; thus, it is difficult to assess whether levels were changed on an individual basis. Fourth, the duration of hypertension and antihypertensive treatment could have a role in influencing the association between the use of ACEI and EPC mobilization. However, the actual duration of hypertension in our patients was impossible to determine. Similarly, data regarding the length of ACEI therapy could not be reliably determined. Furthermore, we measured the number of EPCs, but not their function; however, numbers and functional integrity are coregulated by the same molecular pathways, and therefore, a decrease in EPC numbers is usually associated with a corresponding decrease in function and vice versa.

## Figures and Tables

**Table 1:** Clinical characteristics of groups I and II.

Clinical feature	Group I (*n* = 43)	Group II (*n* = 22)	
Qualitative parameters	*n* (%)	*n* (%)	*p* value^∗^
Male gender	20 (46.5)	10 (45.5)	0.9227
Sever clinical status			
NIH ≤ 7 (%)	17 (39.6)	—	
NIH 8–17	13 (30.2)	—	
NIH > 18	13 (30.2)	—	
Neurological status improvement			
No improvement	16 (37.2)	—	
Improvement < 50%	13 (30.2)	—	
Improvement > 50% or complete recovery	14 (32.6)	—	
Death	3 (7.0)	—	
Hypertension	43 (100)	20 (90.9)	0.2100
Dyslipidemia	17 (39.6)	7 (31.8)	0.5419
Diabetes mellitus	15 (34.9)	6 (27.2)	0.5347
Current smoking	8 (18.6)	4 (18.2)	0.9328
Excessive alcohol intake	8 (18.6)	3 (13.6)	0.5858
Obesity	11 (25.6)	4 (18.2)	0.5029
Overweight	20 (46.5)	7 (31.8)	0.2553
Coronary artery disease	23 (53.5)	7 (31.8)	0.0973
Atrial fibrillation	16 (37.2)	6 (27.2)	0.4231
ACE inhibitors	33 (76.7)	13 (59.1)	0.1387
Aspirin	18 (41.9)	8 (36.4)	0.1387
Enoxaparin	2 (4.7)	1 (4.5)	0.9847
Quantitative parameters	Mean ± SD	Mean ± SD	*p* value^∗∗^
Age (y)	75.2 ± 11.3	72.6 ± 9.3	0.1553
Plasma hsCRP (mg/L)	8.2 ± 18.3	3.0 ± 7.6	0.0322
Plasma fibrinogen (mg/dL)	378.3 ± 91.5	360 ± 67.6	0.2517
White blood cells (WBC) (G/L)	9.5 ± 3.21	6.6 ± 2.01	0.5723
Plasma total homocysteine (*μ*mol/L)	14.2 ± 8.8	12.0 ± 6.1	0.4969
Lipid profile (mg/dl)			
Total cholesterol	178.2 ± 44.2	176.1 ± 41.1	0.9117
LDL cholesterol	111.0 ± 39.4	101 ± 31.2	0.3494
HDL cholesterol	45.5 ± 13.7	39 ± 11.2	0.1344
Triglycerides	113.3 ± 40.2	99 ± 30.2	0.3050

^∗^Chi-squared test. ^∗∗^Mann–Whitney *U* test.

**Table 2:** Circulating stem cells (CD133^+^) and early EPCs (CD133^+^/VEGFR2^+^) in groups I (at the first time point—24 h) and II.

Study population (Number of cells/*μ*L)	Group I (*n* = 43) Mean ± SD	Group II (*n* = 22) Mean ± SD	*p* value^∗∗^
CD133^+^	0.0325 ± 0.054	0.0162 ± 0.0132	0.6196
CD133^+^/VEGF-R2^+^	0.0099 ± 0.013	0.0106 ± 0.0231	0.6253

^∗∗^Mann–Whitney *U* test.

**Table 3:** Clinical characteristics of groups Ia and Ib.

Clinical feature	Group Ia (*n* = 33)	Group Ib (*n* = 10)	*p* value
Qualitative parameters	*n* (%)	*n* (%)	*p* value^∗^
Male gender	14 (42.4)	6 (60.0)	0.3289
Severe clinical status			
NIH ≤ 7	14 (42.4)	3 (30.0)	0.4815
NIH 8–17	9 (27.3)	4 (40.0)	0.4427
NIH > 18	10 (30.3)	3 (30.0)	0.9854
Neurological status improvement:			
No improvement	11 (33.3)	5 (50.0)	0.9086
Improvement < 50%	10 (30.3)	3 (30.0)	0.4665
Improvement > 50% or complete recovery	12 (36.4)	2 (20.0)	0.0857
Death	2 (6.5)	1 (10.0)	0.7079
Stroke subtypes			
Large artery atherosclerosis	18 (54.5)	6 (60.0)	0.5266
Cardioembolic	3 (9.1)	1 (10.0)	0.6684
Small vessel disease	2 (6.1)	1 (10.0)	0.5579
Other determined etiologies	1 (3.0)	0 (0)	0.4963
Undetermined etiology	9 (27.3)	2 (20.0)	
Hypertension	33 (100)	10 (100)	1.0000
Dyslipidemia	15 (45.5)	2 (20.0)	0.1492
Diabetes mellitus	13 (39.4)	2 (20.0)	0.2596
Current smoker	4 (12.1)	4 (40.0)	0.0547
Excessive alcohol intake	6 (18.1)	2 (20.0)	0.8970
Obesity	9 (27.3)	2 (20.0)	0.7465
Overweight	15 (45.5)	5 (50.0)	0.8989
Coronary heart disease	21 (63.6)	4 (40.0)	0.1844
Atrial fibrillation	13 (39.4)	3 (30.0)	0.5903
ACE inhibitors	33 (100)	0 (0)	—
Aspirin	17 (51.5)	1 (10.0)	0.0197
Enoxaparin	2 (6.1)	0 (0)	0.4253
Thrombolitic therapy	3 (9.1)	3 (30.0)	0.1270
Quantitative parameters	Mean ± SD	Mean ± SD	*p* value^∗∗^
Age	76.2 ± 9.43	71.6 ± 16.06	0.4376
Plasma hsCRP (mg/L)	9.6 ± 20.63	3.4 ± 4.64	0.0223
Plasma fibrinogen (mg/dL)	393.8 ± 83.98	330.6 ± 101.69	0.0478
White blood cells (WBC) (G/L)	9.6 ± 9.81	9.2 ± 7.63	0.8213
Plasma total homocysteine (*μ*mol/L)	14.8 ± 9.77	11.9 ± 3.39	0.7848
Lipid profile (mg/dl)			
Total cholesterol	178.7 ± 45.78	176.6 ± 40.52	0.8181
LDL cholesterol	111.1 ± 40.16	110.9 ± 38.65	0.9313
HDL cholesterol	45.5 ± 14.26	45.4 ± 12.44	0.8518
Triglycerides	109.4 ± 36.3	126.2 ± 51.05	0.3652

^∗^Chi-squared test. ^∗∗^Mann–Whitney *U* test.

**Table 4:** Circulating stem cells (CD133^+^) on subsequent days (1, 3, and 7) of AIS in groups Ia and Ib.

Study population (Number of cells/*μ*L)	Group Ia (*n* = 33) Mean ± SD	Group Ib (*n* = 10) Mean ± SD
CD133^+^_1	0.0331 ± 0.07	0.0194 ± 0.02
CD133^+^_3	0.0171 ± 0.01	0.0195 ± 0.01
CD133^+^_7	0.0162 ± 0.01	0.0257 ± 0.01

**Table 5:** Circulating early EPCs (CD133^+^/VEGFR2^+^) on subsequent days (1, 3, and 7) of AIS in group Ia.

Study population (Number of cells/*μ*L)	Group Ia (*n* = 33) Mean ± SD	Group Ib (*n* = 10) Mean ± SD
CD133^+^/VEGF-R2^+^_1	0.0103 ± 0.02	0.0062 ± 0.007
CD133^+^/VEGF-R2^+^_3	0.0044 ± 0.03	0.0064 ± 0.005
CD133^+^/VEGF-R2^+^_7	0.0033 ± 0.001	0.0102 ± 0.006
