# Erratum to “Effects of Angiotensin-Converting Enzyme Inhibition on Circulating Endothelial Progenitor Cells in Patients with Acute Ischemic Stroke”

**DOI:** 10.1155/2020/7946807

**Published:** 2020-11-04

**Authors:** Monika Gołąb-Janowska, Edyta Paczkowska, Bogusław Machaliński, Dariusz Kotlęga, Agnieszka Meller, Krzysztof Safranow, Michał Maj, Przemysław Nowacki

**Affiliations:** ^1^Department of Neurology, Pomeranian Medical University, Szczecin, Poland; ^2^Department of General Pathology, Pomeranian Medical University, Szczecin, Poland; ^3^Department of Biochemistry and Medical Chemistry, Pomeranian Medical University, Szczecin, Poland

In the article titled “Effects of Angiotensin-Converting Enzyme Inhibition on Circulating Endothelial Progenitor Cells in Patients with Acute Ischemic Stroke” [1], additional references should have been cited, included in the text below as references 61-68 [2–9].

The details of where these references should have each been cited are as follows:

*Reference 61*. In Page 2, at the end of first paragraph, after the sentence “In addition, assessing endothelial damage is important when evaluating EPC levels [5].”

*References 62, 63*. In Page 6, at the last paragraph of the first column, after the sentence “This occurs in patients with acute coronary syndrome and acute ischemic stroke, with a peak in EPC counts and vascular endothelial growth factor(VEGF) levels occurring on the seventh day after the ischemic event [16, 38–40].”

*Reference 64*. In Page 6, at the first paragraph of the second column, after the sentence “In previous studies involving patients with myocardial infarction, a higher number of EPCs were associated with better prognosis, increased myocardial salvage, and more collateral in the ischemic zone [41, 42].”

*References 65-68*. In Page 7, at the sixth line of the second column, after “Indeed, Mandraffino et al. [60].”
